# Rotational Mode-Specificity in the Cl + C_2_H_6_ → HCl + C_2_H_5_ Reaction

**DOI:** 10.1021/acs.jpca.2c01526

**Published:** 2022-04-15

**Authors:** Dóra Papp, Gábor Czakó

**Affiliations:** MTA-SZTE Lendület Computational Reaction Dynamics Research Group, Interdisciplinary Excellence Centre and Department of Physical Chemistry and Materials Science, Institute of Chemistry, University of Szeged, Rerrich Béla tér 1, Szeged H-6720, Hungary

## Abstract

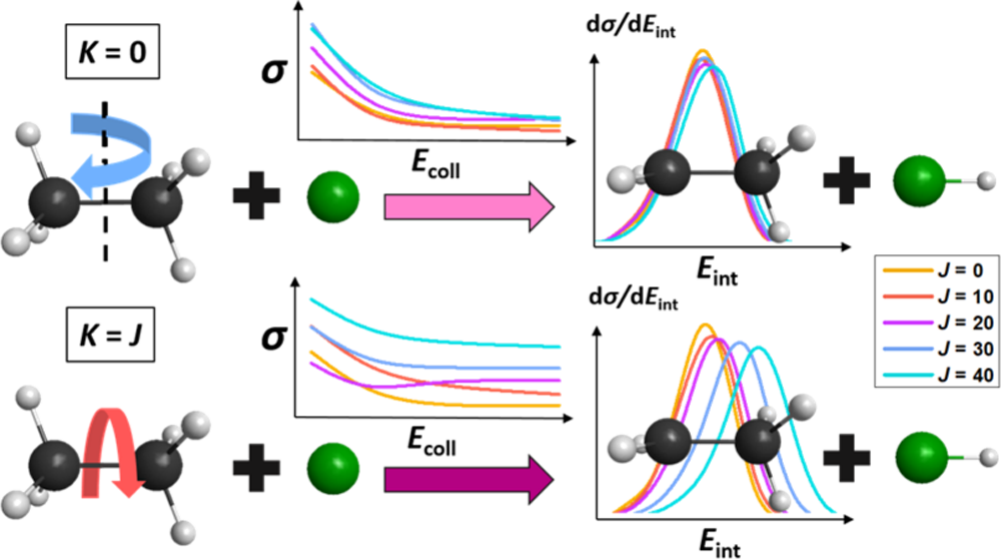

We perform rotational mode-specific quasi-classical trajectory simulations using a high-quality ab initio analytical potential energy surface for the Cl(^2^P_3/2_) + C_2_H_6_ → HCl + C_2_H_5_ reaction. As ethane, being a prolate-type symmetric top, can be characterized by the *J* and *K* rotational quantum numbers, the excitation of two rotational modes, the tumbling (*J*, *K* = 0) and spinning (*J*, *K* = *J*) rotations of the reactant is carried out with *J* = 10, 20, 30, and 40 at a wide range of collision energies. The impacts of rotational excitation on the reactivity, the mechanism, and the post-reaction distribution of energy are investigated: (1) exciting both rotational modes enhances the reactivity with the spinning rotation being more effective due to its coupling to the C–H stretching vibrational normal modes (C–H bond elongating effect) and larger rotational energies, (2) rotational excitation increases the dominance of direct rebound over the stripping mechanism, while collision energy favors the latter, (3) investing energy in tumbling rotation excites the translational motion of the products, while the excess spinning rotational energy readily flows into the internal degrees of freedom of the ethyl radical or, less significantly, into the HCl vibration, probably due to the pronounced rovibrational coupling in this case. We also study the relative efficiency of vibrational and rotational excitation on the reactivity of the barrierless and thus translationally hindered title reaction.

## 1 Introduction

The relative efficiency of different forms of the energy invested in a chemical reaction has been widely studied both experimentally and theoretically in the past decades. For the smallest A + BC atom + diatom reactions, the first rules of thumb of energy efficiency have been established:^1^ translational energy promotes such a reaction if the reactants have to surmount an early barrier, that is, the reaction has a reactant-like transition state (TS), while vibrational energy enhances a late-barrier reaction having a product like TS structure with an elongated B–C bond. In the case of more complex chemical reactions involving a polyatomic molecule, the question of mode-specificity naturally rises: which vibrational mode of the polyatomic reactant should be excited to promote (or inhibit) the reaction? Or, even more specifically, how can one reach the selective cleavage of a chemical bond? These issues have also been extensively investigated,^2−25^ involving even 7- and 9-atomic systems, as well.^26−33^

However, a somewhat less studied phenomenon is when the rotational motion of a polyatomic reactant molecule is excited. In atom + diatom reactions, where the rotation of the diatom can be characterized by the *J* rotational quantum number, rotational excitation effects have been the focus of several experimental, classical, and quantum dynamics studies.^34−41^ Stepping forward, in the case of polyatomic reactants, rotational mode-specificity can also be defined and investigated, as a symmetric/asymmetric polyatomic rotor can also be characterized by *K*/*K*_a_*K*_c_ quantum number/labels, the projections of the **J** total rotational angular momentum to the body-fixed axes. Thus, the *K*/*K*_a_*K*_c_ quantum number/labels, which can adopt values in the [0, ±1, ..., ±*J*] interval, necessarily introduce rotational mode-specificity as their different values correspond to different rotational modes (states) of the reactant molecule. Accordingly, rotational mode-specific studies for the H_2_O^+^ + H_2_/D_2_,^42,43^ H/F/Cl + H_2_O,^44−46^ F/Cl/OH + CH_4_,^47−50^ H/Cl/O + CHD_3_,^51−54^ and the F^–^ + CH_3_F/CH_3_Cl/CH_3_I^55,56^ reactions involving asymmetric, spherical, and symmetric top polyatomic reactants have been carried out.

Experiments found significant rotational promotion in the case of the H_2_O^+^(*J*, *K*_a_, *K*_c_) + D_2_ reaction,^42^ which was later explained by simulations as the facilitated reorientation of the H_2_O^+^ molecule due to rotational excitation.^43^ In the case of the H + CHD_3_ reaction, a seven-dimensional quantum dynamics study found basically no effect of rotational excitation up to *J* = 2;^51^ however, for Cl + CHD_3_(*v*_1_ = 1) → HCl + CD_3_, a joint crossed-beam, quasi-classical, and quantum dynamics investigation showed that the tumbling rotation (*J*, *K* = 0) of CHD_3_ significantly enhances the reactivity, while the spinning rotation of the reactant around the C–H axis (*K* = *J*) has only a minor effect.^52^ It was shown for this,^52^ and also for the O(^3^P) + CHD_3_(*v*_1_ = 0,1) → OH + CD_3_,^54^ reaction that rotational excitation does not affect the scattering angle distribution of the products, but the initial attack angle distributions indicated the enlargement of the reactive cone of acceptance with increasing *J*. Interestingly, for the OH + CH_3_ → O + CH_4_ reaction, quantum dynamics simulations found that the rotational excitation of both of reactants hinders the reaction.^57,58^ Rotational mode-specific computations involving the F^–^ + CH_3_F/CH_3_Cl bimolecular nucleophilic substitution (S_N_2) reactions observed substantial rotational inhibition as well.^55^ In the former case, both the spinning (*K* = *J*) and the tumbling (*K* = 0) rotation of the CH_3_F reactant had a similar inhibiting effect, whereas for the latter reaction, the tumbling rotation was found to be less effective in hindering reactivity.^55^ Very recently, the F^–^ + CH_3_I reaction was also studied and showed considerable rotational hindrance in the case of the S_N_2 channel for both tumbling and spinning excitations, whereas the proton-transfer reaction was noticeably promoted with increasing *J* by exciting the spinning reactant rotational mode; however, it was left unaffected by tumbling excitation.^56^

In the present work, we study the effect of rotational excitations for a 8-atomic reactant molecule, namely, ethane, in the Cl(^2^P_3/2_) + C_2_H_6_ → HCl + C_2_H_5_ reaction by performing quasi-classical trajectory (QCT) simulations on our recently developed high-quality ab initio full-dimensional potential energy surface (PES).^59^ Ethane is a prolate-type symmetric top, characterized by the *J* and *K* rotational quantum numbers, and we focus on the two limiting cases, (1) *K* = 0, referring to the rotation around the axis perpendicular to the C–C bond, that is *tumbling* rotation, and (2) *K* = ±*J*, denoting the *spinning* rotation around the C–C bond. We investigate the different rotational-mode excitations on the reactivity, the mechanism, and the energy flow during the reactions. We also compare the relative efficacy of rotational, translational, and even vibrational^31^ form of energy investments.

## 2 Methods

In the Cl + C_2_H_6_ → HCl + C_2_H_5_ reaction, the reactant ethane molecule within the rigid-rotor approximation is a prolate-type symmetric top; thus, it can be characterized by two rotational quantum numbers, *J* and *K*. The *J* quantum number is related to the *j* length of the **j** classical total angular momentum vector as
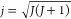
1where *j* is
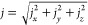
2where *j*_α_ (α = *x*, *y*, *z*) are the three components of the **j** total angular momentum vector in the body-fixed coordinate system (principal axis system) and the *K* quantum number is identified as the projection of **j** on the body-fixed *x* axis, that is, *K* = *j*_*x*_ (this latter convention applies only for the prolate-type case).

During the QCT simulations, after setting *j*_*x*_ to the *K* quantum number and *j* according to eq 1, *j*_*y*_ and *j*_*z*_ are randomly sampled based on the expressions
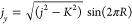
3
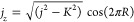
4where *R* ∈ [0,1] is a real random number. Then, the **j** vector is transformed to the space-fixed Cartesian coordinate system defined in the QCT computations. The initial angular momentum is tuned by standard modifications of the **v**_*i*_ velocity vector of each atom^60^
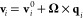
5where
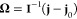
6where **Ω** is the angular velocity vector, **q**_*i*_ are the Cartesian coordinates of the *i*th atom, **I** is the moment of inertia tensor, and **j**_0_ is the preexisting angular momentum vector corresponding to the **v**_*i*_^0^ initial velocities.

In quantum dynamics, the initial *JK*-specific state of the reactant can also be selected as was done in ref (52) in the case of the Cl + CHD_3_ reaction. In QCT simulations, an assembly of trajectories with different *R* values (**j** directions, precessing around the principal axis) corresponds to a specific initial rotational quantum state.

## 3 Computational Details

We carry out quasi-classical computations using our recently developed PES^59^ for the Cl + C_2_H_6_ → HCl + C_2_H_5_ reaction by applying different rotational excitations of the reactant ethane molecule. We examine the two limiting cases of rotational mode-, that is, *K*-quantum-number dependency: the *K* = 0 and the *K* = *J* cases, while the *J* quantum number adopts the following values: 0, 10, 20, 30, and 40. These *J*, *K* pairs correspond to 0.21, 0.80, 1.76, and 3.11 kcal/mol rotational energies when *K* = 0 and to 0.79, 3.11, 6.96, and 12.36 kcal/mol rotational energies when *K* = *J* for *J* = 10, 20, 30, and 40, respectively.

The initial orientation of the reactants is random, and the initial vibrational state, the zero-point energy (ZPE), of ethane is set using standard normal-mode sampling.^60^ The initial distance of the reactants is , where *x* = 16 bohr and the *b* impact parameter is varied between 0 and *b*_max_ (where the reactivity vanishes) with a step size of 1 bohr. The simulations are carried out at the following collision energies: 1.0, 3.0, 5.5, and 10.0 kcal/mol. We run 500 trajectories for each *b*–*J*, *K* pair−collision energy combination. The setting of the initial *J* and *K* quantum numbers of ethane is described in Section 2 in detail.

The integral cross sections (ICSs) for the title reaction at different collision energies and for different *J*, *K* settings are calculated by using a *b*-weighted numerical integration of the *P*(*b*) opacity functions (the reaction probabilities as a function of the impact parameter). We apply different ZPE constraints for the ICSs: (1) *soft*: the sum of the classical vibrational energy of the ethyl radical product and the classical internal energy of the HCl product must be larger than the sum of the harmonic ZPE of the ethyl radical and the anharmonic ZPE of HCl corresponding to its actual rotational state. The variationally determined anharmonic rovibrational levels of HCl are taken from ref (6), (2) *hard*: the above constraints are set for each product separately, (3) *ethyl ZPE*: the ZPE constraint is only applied for the ethyl radical. The scattering angle distribution of the products is obtained by binning the cosine of the angle θ of the relative velocity vector of the reactants and that of the products into 5 equidistant bins, where backward scattering corresponds to cos(θ) = −1.

The initial attack angle of the reactants is considered to be the included angle of the initial C–C vector and the initial velocity vector of the center of mass of the ethane molecule. The rotational quantum number of the HCl product is determined as described in detail in ref (59), and the vibrational quantum number of HCl is identified as that of the anharmonic rovibrational energy level nearest to the classical internal energy of HCl corresponding to its rotational state.

As it has been shown previously for the rovibrationally unexcited and the *v*_1_ = 1 vibrationally excited Cl + C_2_H_6_(*v* = 0,*v*_1_ = 1) → HCl + C_2_H_5_ reactions,^31^ where QCT data obtained from 500 trajectories per collision energy—vibrational mode—impact parameter (1 bohr step size) combination were tested against QCT data computed from 1000 trajectories per collision energy—vibrational mode—impact parameter (0.5 bohr step size),^59^ the relative uncertainties of ICSs, product angular distributions, and opacity functions turned out to be less than 10% (4% for the non-constrained ICSs), while the average (maximum) absolute deviations of the reaction probabilities and the hard-constrained HCl vibrational-state probabilities were 0.01 (0.04) and 0.03 (0.07), respectively.

## 4 Results and Discussion

**Figure 1:**
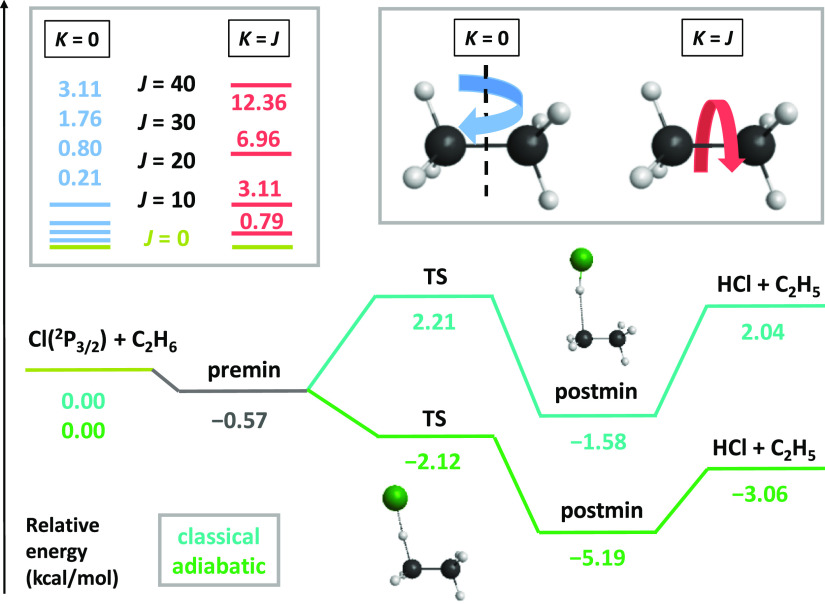
Schematic representation of the classical and adiabatic PES^59^ of the Cl(^2^P_3/2_) + C_2_H_6_(*J*, *K*) → HCl + C_2_H_5_ reaction including rotational energy levels for different *J* values when *K* = 0 (tumbling rotation) and when *K* = *J* (spinning rotation) determined on the PES.

We perform rotational mode-specific (QCT) computations for the Cl(^2^P_3/2_) + C_2_H_6_(*J*, *K*) → HCl + C_2_H_5_ reaction using our recent full-dimensional analytical PES fitted on high-quality spin-orbit-corrected ab initio energy points applying the monomial symmetrization approach^61^ and developed by using the Robosurfer program system.^62^ The PES, whose energetics is shown in Figure 1, features a slightly late barrier for the reaction, with a classical/adiabatic height of 2.21/–2.12 kcal/mol, and the reaction has a classical/adiabatic 2.04/–3.06 kcal/mol endothermicity/exothermicity, with a shallow entrance-channel well and a relatively deep exit-channel minimum. Thus, with the ZPE-correction included, the title reaction is barrierless and exothermic; however, the H-abstraction process covers only about a 5 kcal/mol energy range. Rotational excitation energies, calculated as
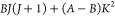
7where *A* ≫ *B* are the non-equal rotational constants of ethane determined on the PES, are 0.21, 0.80, 1.76, and 3.11 kcal/mol corresponding to *J*, *K* = 0 (tumbling rotation) and are 0.79, 3.11, 6.96, and 12.36 kcal/mol when *K* = *J* (spinning rotation) for *J* = 10, 20, 30, and 40, respectively. Due to the smaller moment of inertia in the *K* = *J* case, based on the
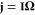
8expression, the same *J* quantum number results in larger angular velocity (**Ω**) and therefore larger rotational energy. The *K* = *J* energies are commeasurable with some of the fundamental vibrational energies of ethane;^31^ thus, their effect on the reaction can be readily compared.

**Figure 2:**
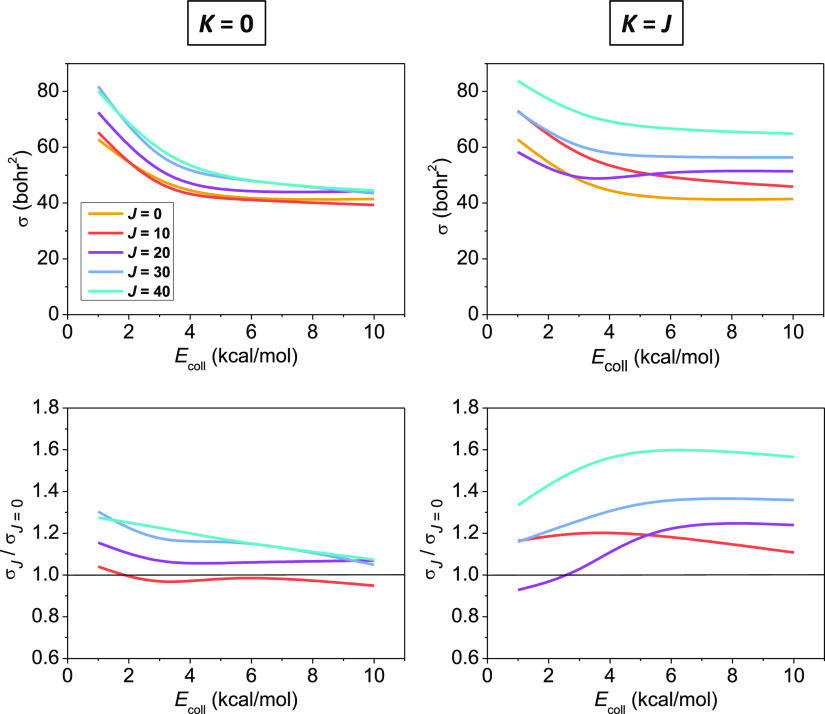
ICSs of the Cl + C_2_H_6_(*J*, *K*) → HCl + C_2_H_5_ reaction and their enhancement factors relative to the rotationally unexcited reaction as a function of collision energy for different *J*, *K* pairs.

We run trajectories for different rotational quantum numbers (*J* and *K*) of the prolate-type symmetric top reactant ethane molecule at collision energies 1.0, 3.0, 5.5, and 10.0 kcal/mol. As seen in Figure 2, rotational excitation promotes the title reaction in almost all cases, slight inhibition can only be observed for *J* = 10, *K* = 0, and *J* = *K* = 20. It is also clear from Figure 2 that higher *J* values usually enhance reactivity more and more for both rotational modes. The shape of the excitation functions, that is, the ICSs as a function of collision energy, is not affected significantly by rotational excitation, however, a slightly faster decay can be seen at higher *J* values when *K* = 0, except for the *J* = *K* = 20, where an interesting drop in the cross section at low collision energies can be observed. The decaying shape of the excitation function is originated from the submerged barrier, which makes reaction time itself a promoting effect. The reactivity enhancement due to the spinning rotation excitation is much stronger than that caused by exciting the tumbling mode, which can be explained by the above-mentioned larger angular velocity in the former case, and the corresponding centrifugal force, which may cause a significant elongation of the C–H bonds and thereby facilitates the abstraction of one of the H atoms. A similar observation could be made for the F^–^ + CH_3_I proton-transfer reaction as well.^56^ Also in line with this, exciting the spinning rotation around the C–H bond in the X + CHD_3_ → HX + CD_3_ [X = Cl, O] reactions is found to have only a minor effect on reactivity.^52,54^ In the title reaction, tumbling rotational excitation might rather induce the elongation of the C–C bond, which is almost a spectator during H-abstraction^31^ and only mildly influences the C–H-bond elongation. From the rotational reactivity enhancements relative to the unexcited reaction, shown in Figure 2, the slow decay of the *K* = 0 promotion can be observed with increasing collision energy, whereas exciting the *K* = *J* mode leads to a stronger boosting of the reaction at higher collision energies (except for *J* = 10) with small maxima at 6–8 kcal/mol. Surprisingly, in contrast to vibrational^52^ and tumbling rotational excitation, the impact of the spinning rotational excitations on reactivity strengthens as collision energy increases. This might be explained by the time scales (few tenths of picoseconds) of collisions and the excited spinning rotation (4 times faster than tumbling rotation) approaching each other as collision energy increases, thereby making the rotational effect enhanced. Moreover, probably due to the higher *K* = *J* rotational energies and their higher growth with increasing *J*, exciting spinning rotation enhances reactivity much more effectively than tumbling rotation: in the *K* = *J* case, even a 1.6 increasing factor is reached at *J* = 40.

**Figure 3:**
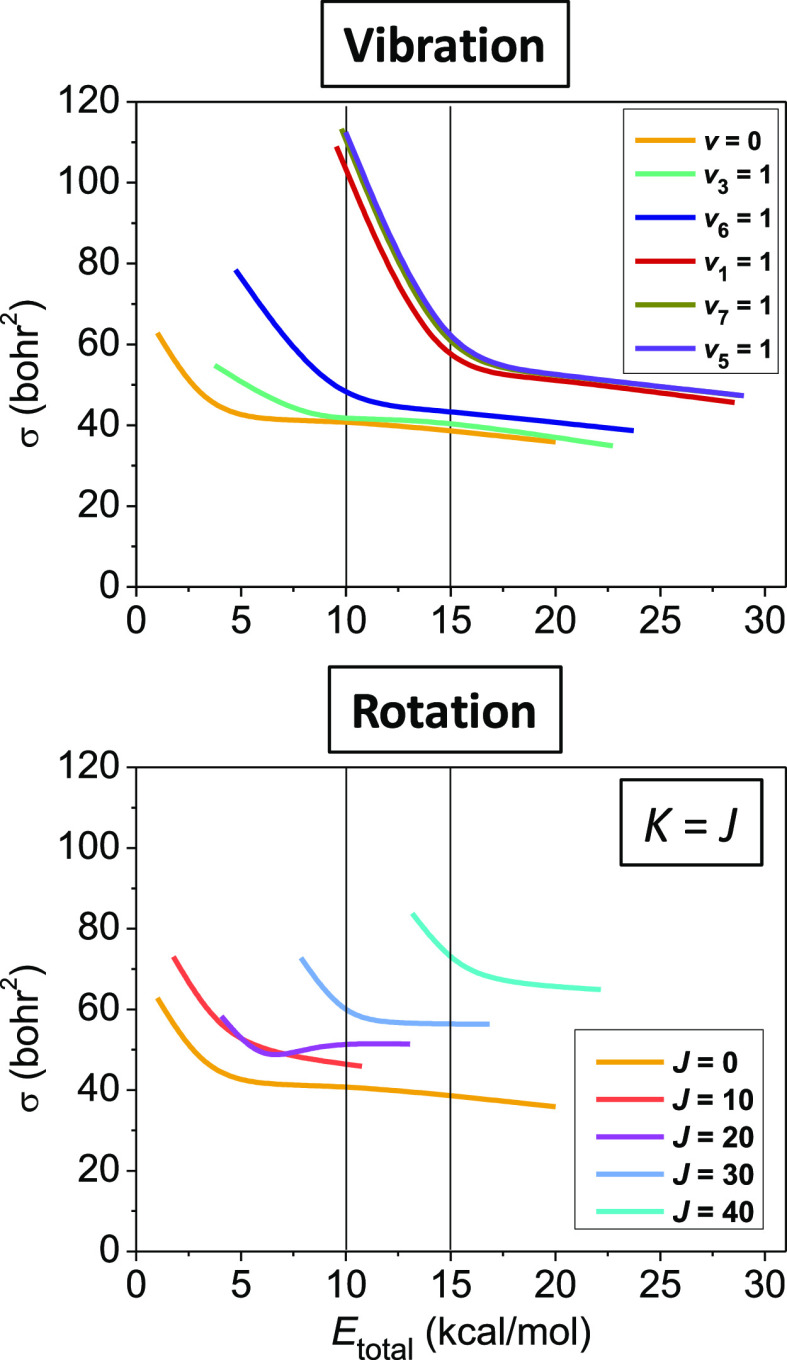
ICSs as a function of total energy for the Cl + C_2_H_6_ → HCl + C_2_H_5_ reaction computed on our PES from ref (59) with C_2_H_6_(*v* = 0) and C_2_H_6_(*v*_*x*_ = 1) [*x* = 1, 3, 5, 6, 7] vibrationally and C_2_H_6_(*J*, *K* = *J*) [*J* = 0, 10, 20, 30, 40] rotationally excited reactants. The unexcited ICS value at a 20 kcal/mol collision energy in the lower panel is taken from ref (31). The upper panel is adapted with permission from ref (31). Copyright 2021 American Institute of Physics.

As to the relative efficiency of rotational and vibrational energy invested in the title reaction, in Figure 3, we plot the ICSs of the title reaction in the case of five different vibrationally^31^ and four different spinning-rotationally excited cases as a function of the total initially available energy (collision energy + vibrational/rotational excitation energy). Note that due to the narrow range of the small rotational energies corresponding to the tumbling rotation, we omit it from this comparison. If we take a look at the panels of Figure 3 at 10 kcal/mol total energy, we can see an almost triple increase in the ICSs relative to the unexcited reaction when the C–H-stretching modes (*v*_*x*_ = 1 [*x* = 1,5,7]) are excited, in which case, 90% of the total energy is in the vibrational degree of freedom. At the same total energy, the *J* = *K* = 20 and 30 rotational excitations lead to approximately 25 and 40% reactivity enhancement with the rotational energy being 30 and 70% of the total energy. Furthermore, at 15 kcal/mol total energy, we observe 50% C–H-stretching-caused (60% of the total energy in the vibrational modes) vibrational and 30/75% *J* = *K* = 30/40 rotational increment (45/80% in rotation) in the ICSs. Based on the above, we can conclude that the vibrational excitation (of the CH-stretching modes) can result in a somewhat more effective reactivity enhancement, especially at low collision energies, than rotational excitation, with the latter being still significant and competitive with the former as collision energy increases.

**Figure 4:**
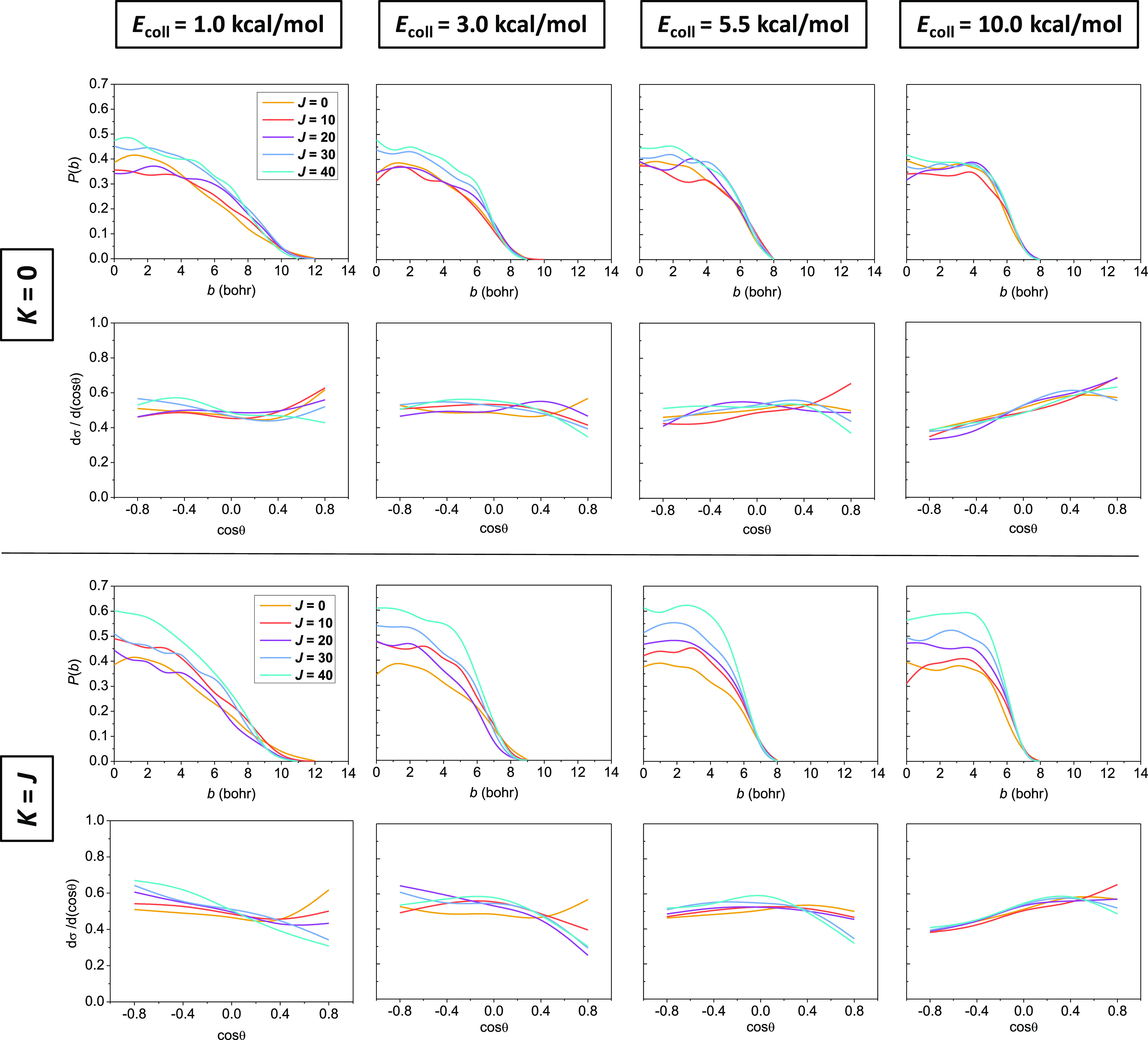
Reaction probabilities (upper rows) and normalized product scattering angle distributions (lower rows) of the Cl + C_2_H_6_(*J*, *K*) → HCl + C_2_H_5_ reaction at different collision energies in the *K* = 0 and *K* = *J* limiting cases for different values of *J*.

From the reaction probabilities and the product scattering angle distributions shown in Figure 4, we can see that rotational enhancement is the most pronounced at small impact parameters since the *b*_max_ values do not change considerably upon the rotational excitation of ethane, while the probabilities rise significantly with increasing *J* at smaller *b* values. This results in a small drop in the otherwise basically isotropic (except for the highest collision energy where the products are mainly forward scattered) angular distributions near cos(θ) = 1 with increasing *J* for both tumbling and spinning rotations, which indicates a slightly increased backward scattering as *J* increases, the signature of the direct rebound mechanism, dominant at small *b* values. As collision energy increases, the stripping mechanism, occurring at larger impact parameters, becomes prevalent, causing the clearly forward scattered pattern at 10 kcal/mol (dropping also at cos(θ) = 1 with increasing *J*). The more intense promoting effect of the spinning excitations is also clear from the reaction probabilities compared to the tumbling rotation, and therefore, the drop in the normalized product angular distributions near the forward direction is also more pronounced in the *K* = *J* case.

If we inspect the distributions of the initial attack angle (α) of the reactants of reactive trajectories, defined as the angle between the initial C–C vector and the initial velocity vector of the center of mass of C_2_H_6_, shown in Figure S1 in the Supporting Information, we cannot observe significant *J*-dependency, similarly to the F^–^ + CH_3_X [X = Cl, I] S_N_2 reactions.^55,56^ However, this finding is in sharp contrast to the behavior of the O/Cl + CHD_3_ reactions^52,54^ upon rotational excitation: in these cases, the tumbling rotation (*K* = 0) enhances the reactivity of H-abstraction by enlarging the reactive cone of acceptance, while the attack angles corresponding to the *K* = *J* excitation (spinning around the C–H axis) are less dependent on *J*. For the title reaction, due to the *D*_3d_ symmetry of ethane, the attack angle distributions are supposed to be symmetric with respect to cos(α) = 0, which is shown in Figure S1, within statistical accuracy. The essentially isotropic distributions suggest that both the front-side (along the *C*_3_ axis) and side-on (along the axis perpendicular to the C–C bond) reactivities are basically the same; however, a very slight preference in the spinning-excited cases can be seen for the perpendicular direction. In the case of spinning rotation, occurring around the C–C bond, the Cl atom approaching the front side of ethane along the *C*_3_ axis finds a H atom with similar probability if *b* is small, independent of the intensity of the rotation, while when it is approaching from the side-on direction, the excited rotational motion of the H atoms around the C–C axis increases the probability of Cl finding a H atom to react with. This phenomenon is basically independent of the change in collision energy and becomes more apparent with increasing *J*.

**Figure 5:**
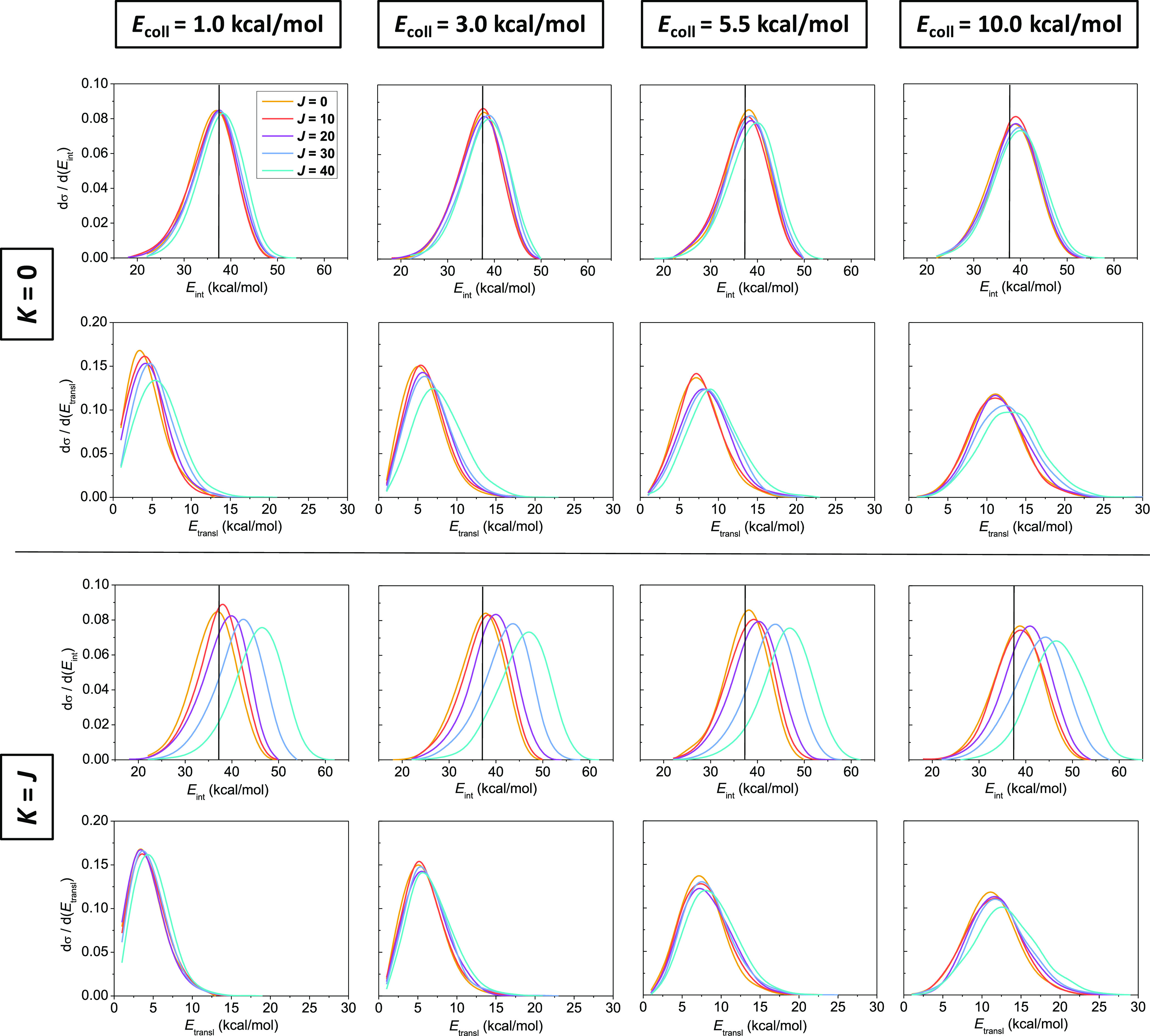
Normalized internal energy distribution of the ethyl radical where vertical black lines refer to the ZPE of the ethyl radical (37.4 kcal/mol) (upper rows) and normalized relative translational energy distributions of the products (lower rows) of the Cl + C_2_H_6_(*J*, *K*) → HCl + C_2_H_5_ reaction at different collision energies in the *K* = 0 and *K* = *J* limiting cases for different values of *J*.

As to the impact of rotational excitation on the energy flow during the title reaction, the difference between the two rotational modes is clearly shown in Figure 5: the excess rotational energy excites the relative translational motion of the products, while the internal energy of the ethyl radical is basically unaffected during tumbling rotation, whereas the energy of the excited spinning rotational mode is mostly fueled into the internal degrees of freedom of C_2_H_5_, while only negligible rotational energy flows into the translational motion in this case. These effects are present at all collision energies (of course, the ones corresponding to the tumbling rotation are smaller due to the smaller rotational energies) and are more and more pronounced as *J* increases. In addition, higher collision energies also induce stronger translational excitation of the products upon the increase of *J* in the case of spinning rotation. Interestingly, in the F^–^ + CH_3_I → HF + CH_2_I^–^ reaction, the spinning rotation around the C–I axis, leading to C–H elongation, gave rise to a hotter translational distribution of the products.^56^ The translational energy distributions of the unexcited reaction widen with increasing collision energy accompanied by only minor changes in the internal energy distributions. The fundamentally different behavior of the two rotational modes in the title reaction might be explained, on one hand, by the stronger coupling of the spinning rotation with the C–H stretching vibrational modes of ethane, and thereby of the ethyl radical product, due to the elongation of the C–H bonds induced by the spinning-generated centrifugal force. At the same time, tumbling rotation basically changes only the relative orientation of the reactants; therefore, the excess energy in this mode might more easily flow into the relative translational degree of freedom of the products. On the other hand, the spinning rotational excitation of ethane is more likely to contribute to the internal energy of the ethyl radical product because the C–C bond does not break during the H-abstraction reaction and the shape of the ethyl radical is not very different from that of ethane (with respect to the spinning rotation), while the force acting due to the tumbling rotational motion on the H atoms causes the recoil of the fragments after reaction. It is also apparent from the internal energy distributions of the ethyl radical that a significant amount of the trajectories is ZPE-violating, which is the reason for applying different ZPE constraints on the cross sections (see Figures S2 and S3 of the Supporting Information).

**Figure 6:**
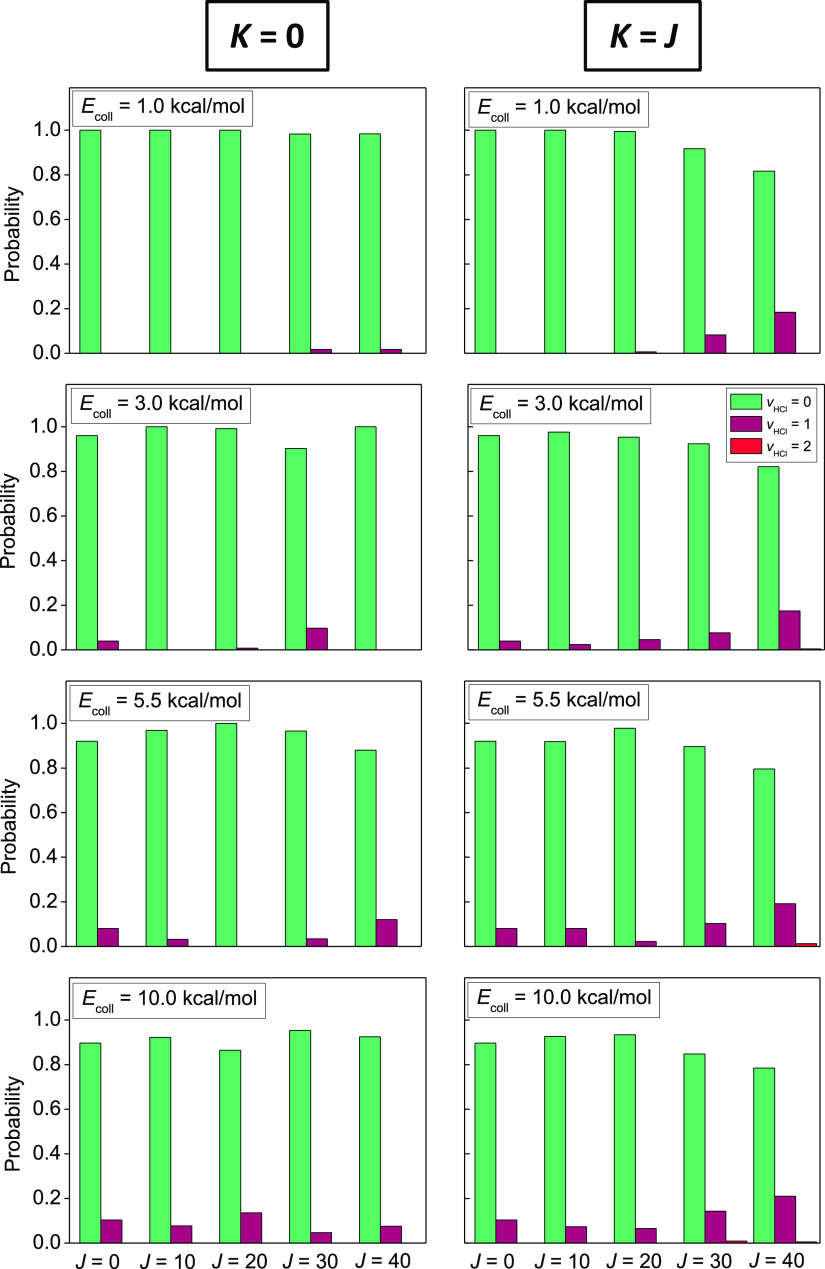
Vibrational state distributions of the HCl product of the Cl + C_2_H_6_(*J*, *K*) → HCl(*v*_HCl_) + C_2_H_5_ reaction at different collision energies in the *K* = 0 and *K* = *J* limiting cases for different values of *J*.

The vibrational state distributions of the HCl product molecule, shown in Figure 6, suggest that the excess energy in the spinning rotation is more likely to excite the HCl vibration at low collision energies, while at the highest 10 kcal/mol energy, this effect is equalized between the spinning and tumbling rotations. The *v*_HCl_ = 1 relative populations increase with increasing *J* due to spinning rotational excitation; even the *v*_HCl_ = 2 state emerges in a few cases, while such a tendency cannot be clearly observed when the tumbling rotation is excited. The rotational influence on the HCl vibration is quite moderate overall, especially compared to vibrational effects, where the CH-stretching excitation energies readily convert into diatomic product vibration in the Cl/F + C_2_H_6_ → HCl/HF + C_2_H_5_ reactions.^31,32^ Translational effects are commeasurable for the title reaction with the rotational ones as the rotationally unexcited reaction also develops some vibrational excitation of HCl as the collision energy increases.

We also applied different ZPE constraints for the products in the case of the different rotational excitations, and their impact on reactivity is shown in Figures S2 and S3 for the tumbling and the spinning rotational cases, respectively. As these figures show, all the applied restrictions provide smaller reactivities than the unrestricted results; nevertheless, the qualitative features of the excitation functions remain similar. The largest changes in magnitude are induced by the hard constraint, where each product must have a larger vibrational energy than its ZPE, with these effects being much clearly present in the case of the tumbling rotations. As seen in Figures 5 and 6, when *K* = *J*, the excess rotational energy remains mostly in the internal motions of the ethyl radical, while the HCl vibration becomes also more excited in this case, which is more and more apparent with increasing *J*; therefore, less trajectories are discarded due to the ZPE constraints, which results in larger increments in reactivity as *J* increases. Accordingly, the rotational effects are especially intensified when the hard constraint is applied: in this case, the spinning rotational enhancement reaches even a factor of 15 at *K* = *J* = 40 at 1 kcal/mol collision energy. An analogous observation can be made for *K* = 0 as well, however, on a smaller scale. The soft and “ethyl ZPE” constraints give very similar results for both rotational modes. From the ICSs plotted as a function of the total available initial energy (collision energy + rotational excitation energy, first row of Figures S2 and S3), the rotational reactivity enhancement is clear, as at a constant total energy, higher *J* values lead to higher reactivity (except for some rare occasions of inhibition).

## 5 Conclusions

We carry out a comprehensive rotational mode-specific study for the negative-barrier Cl(^2^P_3/2_) + C_2_H_6_ → HCl + C_2_H_5_ reaction by performing QCT simulations using our recently developed high-quality ab initio analytic PES. The reactant ethane molecule, being a prolate-type symmetric top, can be characterized by the *J* and *K* rotational quantum numbers within the rigid rotor approximation. We examine the two limiting cases of rotational excitation of ethane: the tumbling (*J*, *K* = 0) and the spinning (*J*, *K* = *J*) rotational modes, with *J* = 10, 20, 30, and 40. We find considerable rotational reaction promotion in almost all cases, with the spinning rotational excitation having a more pronounced impact, on one hand, by making the C–H bonds more and more elongated as *J* increases, and, on the other hand, due to larger rotational energies. The decaying shape of the excitation function (caused by the submerged barrier) of the unexcited reaction does not change significantly upon rotational excitation; however, the rotational effects get stronger with increasing collision energy in the case of spinning excitation, while the enhancement factors decrease with increasing collision energy when tumbling rotation is excited. Comparing the relative efficiency of (spinning) rotational and vibrational excitation on the title reaction, we find that they are commensurable, with vibrational reactivity enhancement, especially in the case of the C–H normal modes, being more significant at low collision energies. Exciting both rotational modes of ethane leads to increased reactivity mainly at small impact parameters (more markedly in the case of spinning rotation), where the backward-scattering direct rebound mechanism is dominant, while increasing collision energy makes the stripping mechanism prevalent, resulting in mostly forward-scattered products. The mainly isotropic initial attack angle distributions show a slight preference for side-on attack for spinning rotational excitations. The excess tumbling rotational energy turns out to flow mainly into the relative translational mode of the products, whereas the energy invested to excite the spinning rotational mode tends to flow into the internal degrees of freedom of the ethyl radical, which might be explained by stronger rovibrational coupling in the latter case (elongated C–H bonds induced by rotational excitation). Rotational excitation has, in general, a minor role in populating the excited vibrational states of the HCl product, falling in the magnitude of translational excitation, and much less significant than vibrational excitation; however, probably due to the above-mentioned rovibrational coupling, the spinning rotational energy can more easily flow to HCl vibration. Since a fair amount of the trajectories is ZPE-violating, we applied three different ZPE restrictions on the ICSs of the title reaction. Finally, we conclude that the present study demonstrates that the reactions of rotationally state-selected polyatomic molecules may open new mode-specific research directions, thereby advancing our fundamental knowledge on chemical reaction dynamics.

## Supporting Information Available

ICSs obtained with various ZPE constraints and initial attack angle distributions (PDF)

## References

[ref1] Polanyi J. C. Some Concepts in Reaction Dynamics. Science 1987, 236, 680–690. 10.1126/science.236.4802.680. 17748308

[ref2] Yoon S.; Holiday R. J.; Crim F. F. Control of Bimolecular Reactivity: Bond-Selected Reaction of Vibrationally Excited CH_3_D with Cl(^2^P_3/2_). J. Chem. Phys. 2003, 119, 4755–4761. 10.1063/1.1591176.

[ref3] Camden J. P.; Bechtel H. A.; Ankeny Brown D. J.; Zare R. N. Comparing Reactions of H and Cl with C–H Stretch-Excited CHD_3_. J. Chem. Phys. 2006, 124, 034311 10.1063/1.2155434. 16438587

[ref4] Yan S.; Wu Y.-T.; Zhang B.; Yue X.-F.; Liu K. Do Vibrational Excitations of CHD_3_ Preferentially Promote Reactivity Toward the Chlorine Atom?. Science 2007, 316, 1723–1726. 10.1126/science.1142313. 17588925

[ref5] Wang F.; Liu K. Enlarging the Reactive Cone of Acceptance by Exciting the C-H Bond in the O(^3^P) + CHD_3_ Reaction. Chem. Sci. 2010, 1, 126–133. 10.1039/c0sc00186d.

[ref6] Czakó G.; Bowman J. M. Dynamics of the Reaction of Methane with Chlorine Atom on an Accurate Potential Energy Surface. Science 2011, 334, 343–346. 10.1126/science.1208514. 22021853

[ref7] Zhang Z.; Zhou Y.; Zhang D. H.; Czakó G.; Bowman J. M. Theoretical Study of the Validity of the Polanyi Rules for the Late-Barrier Cl + CHD_3_ Reaction. J. Phys. Chem. Lett. 2012, 3, 3416–3419. 10.1021/jz301649w. 26290965

[ref8] Liu R.; Yang M.; Czakó G.; Bowman J. M.; Li J.; Guo H. Mode Selectivity for a “Central” Barrier Reaction: Eight-Dimensional Quantum Studies of the O(^3^P) + CH_4_ → OH + CH_3_ Reaction on an Ab Initio Potential Energy Surface. J. Phys. Chem. Lett. 2012, 3, 3776–3780. 10.1021/jz301735m. 26291110

[ref9] Czakó G.; Liu R.; Yang M.; Bowman J. M.; Guo H. Quasiclassical Trajectory Studies of the O(^3^P) + CX_4_(*v*_*k*_ = 0, 1) → OX(*v*) + CX_3_(*n*_1_*n*_2_*n*_3_*n*_4_) [X = H and D] Reactions on an Ab Initio Potential Energy Surface. J. Phys. Chem. A 2013, 117, 6409–6420. 10.1021/jp4038107. 23808940

[ref10] Czakó G. Communication: Direct Comparison Between Theory and Experiment for Correlated Angular and Product-State Distributions of the Ground-State and Stretching-Excited O(^3^P) + CH_4_ Reactions. J. Chem. Phys. 2014, 140, 231102 10.1063/1.4884387. 24952515

[ref11] Yan W.; Meng F.; Wang D. Quantum Dynamics Study of Vibrational Excitation Effects and Energy Requirement on Reactivity for the O + CD_4_/CHD_3_ → OD/OH + CD_3_ Reactions. J. Phys. Chem. A 2013, 117, 12236–12242. 10.1021/jp4090298. 24152064

[ref12] Espinosa-Garcia J. Quasi-Classical Trajectory Study of the Vibrational and Translational Effects on the O(^3^P) + CD_4_ Reaction. J. Phys. Chem. A 2014, 118, 3572–3579. 10.1021/jp502414e. 24786320

[ref13] Welsch R.; Manthe U. Communication: Ro-Vibrational Control of Chemical Reactivity in H + CH_4_ → H_2_ + CH_3_: Full- Dimensional Quantum Dynamics Calculations and a Sudden Model. J. Chem. Phys. 2014, 141, 051102 10.1063/1.4891917. 25106559

[ref14] Jiang B.; Guo H. Control of Mode/Bond Selectivity and Product Energy Disposal by the Transition State: X + H_2_O (X = H, F, O(^3^P), and Cl) Reactions. J. Am. Chem. Soc. 2013, 135, 15251–15256. 10.1021/ja408422y. 24044369

[ref15] Song H.; Xie W.; Zhang C.; Yang M. Toward a Comprehensive Understanding of Mode-Specific Dynamics of Polyatomic Reactions: A Full-Dimensional Quantum Dynamics Study of the H + NH_3_ Reaction. J. Phys. Chem. A 2022, 126, 663–669. 10.1021/acs.jpca.1c08399. 35080397

[ref16] Zhang D.; Yang J.; Chen Z.; Chen R.; Jiang B.; Dai D.; Wu G.; Zhang D.; Yang X. Stretching Excitation Promotes its Cleavage in the F + CHD_3_(*v*_1_ = 1) → HF + CD_3_ Reaction at Low Collision Energies. Phys. Chem. Chem. Phys. 2017, 19, 13070–13074. 10.1039/c7cp01428g. 28484767

[ref17] Fu B.; Shan X.; Zhang D. H.; Clary D. C. Recent Advances in Quantum Scattering Calculations on Polyatomic Bimolecular Reactions. Chem. Soc. Rev. 2017, 46, 7625–7649. 10.1039/c7cs00526a. 29143835

[ref18] Li J.; Jiang B.; Guo H. Reactant Vibrational Excitations Are More Effective than Translational Energy in Promoting an Early-Barrier Reaction F + H_2_O → HF + OH. J. Am. Chem. Soc. 2013, 135, 982–985. 10.1021/ja311159j. 23301908

[ref19] Song H.; Guo H. Mode specificity in bond selective reactions F + HOD → HF + OD and DF + OH. J. Chem. Phys. 2015, 142, 174309 10.1063/1.4919666. 25956102

[ref20] Zhao B.; Sun Z.; Guo H. State-to-State Mode Specificity: Energy Sequestration and Flow Gated by Transition State. J. Am. Chem. Soc. 2015, 137, 15964–15970. 10.1021/jacs.5b11404. 26613942

[ref21] Li J.; Corchado J. C.; Espinosa-Garcia J.; Guo H. Final state-resolved mode specificity in HX + OH → X + H_2_O (X = F and Cl) reactions: A quasi-classical trajectory study. J. Chem. Phys. 2015, 142, 084314 10.1063/1.4913522. 25725738

[ref22] Li J.; Song H.; Guo H. Insights into the bond-selective reaction of Cl + HOD(*n*_OH_) → HCl + OD. Phys. Chem. Chem. Phys. 2015, 17, 4259–4267. 10.1039/c4cp05165c. 25571941

[ref23] Lu D.; Qi J.; Yang M.; Behler J.; Song H.; Li J. Mode specific dynamics in the H_2_ + SH → H + H_2_S reaction.. Phys. Chem. Chem. Phys. 2016, 18, 29113–29121. 10.1039/c6cp05780b. 27730236

[ref24] Song H.; Yang M. Understanding mode-specific dynamics in the local mode representation. Phys. Chem. Chem. Phys. 2018, 20, 19647–19655. 10.1039/c8cp03240h. 30014087

[ref25] Liu Y.; Song H.; Xie D.; Li J.; Guo H. Mode Specificity in the OH + HO_2_ → H_2_O + O_2_ Reaction: Enhancement of Reactivity by Exciting a Spectator Mode. J. Am. Chem. Soc. 2020, 142, 3331–3335. 10.1021/jacs.9b12467. 32011872

[ref26] Alex Kandel S.; Peter Rakitzis T.; Lev-On T.; Zare R. N. Dynamical Effects of Reagent Vibrational Excitation in the Cl + C_2_H_6_(*v*_5_ = 1) → HCl + C_2_H_5_ Reaction. Chem. Phys. Lett. 1997, 265, 121–128. 10.1016/s0009-2614(96)01421-2.

[ref27] Corchado J. C.; Chamorro M. G.; Rangel C.; Espinosa-Garcia J. State-to-State Dynamics of the Cl(^2^P) + C_2_H_6_(*v*_5_, *v*_1_ = 0, 1) → HCl(*v*′, *j*′) + C_2_H_5_ Hydrogen Abstraction Reactions. Theor. Chem. Acc. 2019, 138, 26 10.1007/s00214-019-2416-3.

[ref28] Lu D.; Li J. Mode Specificity of a Multi-Channel Reaction Prototype: F + CH_3_OH → HF + CH_3_O/CH_2_OH. Theor. Chem. Acc. 2020, 139, 157 10.1007/s00214-020-02671-3.

[ref29] Liu Y.; Li J. Quantitative Dynamics of the N_2_O + C_2_H_2_ → Oxadiazole Reaction: A Model for 1,3-Dipolar Cycloadditions. ACS Omega 2020, 5, 23343–23350. 10.1021/acsomega.0c03210. 32954185 PMC7496009

[ref30] Lu D.; Li J.; Guo H. Comprehensive Investigations of the Cl + CH_3_OH→HCl + CH_3_O/CH_2_OH Reaction: Validation of Experiment and Dynamic Insights. CCS Chem. 2020, 2, 882–894. 10.31635/ccschem.020.202000195.

[ref31] Papp D.; Li J.; Guo H.; Czakó G. Vibrational Mode-Specificity in the Dynamics of the Cl + C_2_H_6_ → HCl + C_2_H_5_ Reaction. J. Chem. Phys. 2021, 155, 114303 10.1063/5.0062677. 34551541

[ref32] Papp D.; Czakó G. Vibrational Mode-Specific Dynamics of the F(^2^P_3/2_) + C_2_H_6_ → HF + C_2_H_5_ Reaction. J. Chem. Phys. 2021, 155, 154302 10.1063/5.0069658. 34686045

[ref33] Gao D.; Wang D. Time-Dependent Quantum Dynamics Study of the F + C_2_H_6_ → HF + C_2_H_5_ Reaction. Phys. Chem. Chem. Phys. 2021, 23, 26911–26918. 10.1039/d1cp04212b. 34825679

[ref34] Zhang J.; Dai D.; Wang C. C.; Harich S. A.; Wang X.; Yang X.; Gustafsson M.; Skodje R. T. State to State to State Dynamics of the D + H_2_ → HD + H Reaction: Control of Transition-State Pathways via Reagent Orientation. Phys. Rev. Lett. 2006, 96, 093201 10.1103/PhysRevLett.96.093201. 16606261

[ref35] Barg G. D.; Mayne H. R.; Toennies J. P. Quasiclassical Trajectory Studies of the H + H_2_ Reaction on an Accurate Potential Energy Surface. II. Effect of Initial Vibration and Rotation on Reactivity. J. Chem. Phys. 1981, 74, 1017–1025. 10.1063/1.441234.

[ref36] Aoiz F. J.; Herrero V. J.; Sáez Rábanos V. Effects of Translational, Rotational, and Vibrational Energy on the Dynamics of the D + H_2_ Exchange Reaction. A Classical Trajectory Study. J. Chem. Phys. 1991, 94, 7991–8007. 10.1063/1.460133.

[ref37] Zhang D. H.; Lee S.-Y. Effects of Reagent Rotation on the Dynamics of the H_2_ + OH Reaction: A Full Dimension Quantum Study. J. Chem. Phys. 1998, 109, 2708–2716. 10.1063/1.476881.

[ref38] Zhang D. H.; Lee S.-Y. Effects of Reagent Rotation and the Accuracy of the Centrifugal Sudden Approximation in the H_2_ + CN Reaction. J. Chem. Phys. 2000, 112, 203–211. 10.1063/1.480572.

[ref39] Sukiasyan S.; Meyer H.-D. On the Effect of Initial Rotation on Reactivity. A Multi-Configuration Time-Dependent Hartree (MCTDH) Wave Packet Propagation Study on the H + D_2_ and D + H_2_ Reactive Scattering Systems. J. Phys. Chem. A 2001, 105, 2604–2611. 10.1021/jp003767m.

[ref40] Hayes M. Y.; Deskevich M. P.; Nesbitt D. J.; Takahashi K.; Skodje R. T. A Simple Picture for the Rotational Enhancement of the Rate for the F + HCl → HF + Cl Reaction: A Dynamical Study Using a New Ab Initio Potential Energy Surface. J. Phys. Chem. A 2006, 110, 436–444. 10.1021/jp0535745. 16405315

[ref41] Bulut N.; Kłos J.; Alexander M. H. Accurate Quantum Wave Packet Calculations for the F + HCl → Cl + HF Reaction on the Ground 1^2^A′ Potential Energy Surface. J. Chem. Phys. 2012, 136, 104304 10.1063/1.3692328. 22423835 PMC4108641

[ref42] Xu Y.; Xiong B.; Chang Y. C.; Ng C. Y. Communication: Rovibrationally Selected Absolute Total Cross Sections for the Reaction H_2_O^+^(*X*^2^*B*_1_; *v*_1_^+^*v*_2_^+^*v*_3_^+^ = 000; *N*^+^_*K*a+*K*c+_) + D_2_: Observation of the Rotational Enhancement Effect. J. Chem. Phys. 2012, 137, 241101 10.1063/1.4773099. 23277921

[ref43] Li A.; Li Y.; Guo H.; Lau K.-C.; Xu Y.; Xiong B.; Chang Y.-C.; Ng C. Y. Communication: The Origin of Rotational Enhancement Effect for the Reaction of H_2_O^+^ + H_2_ (D_2_). J. Chem. Phys. 2014, 140, 011102 10.1063/1.4861002. 24410213

[ref44] Jiang B.; Li J.; Guo H. Effects of Reactant Rotational Excitation on Reactivity: Perspectives from the Sudden Limit. J. Chem. Phys. 2014, 140, 034112 10.1063/1.4861668. 25669368

[ref45] Jiang B.; Xie D.; Guo H. Calculation of Multiple Initial State Selected Reaction Probabilities from Chebyshev Flux-Flux Correlation Functions: Influence of Reactant Internal Excitations on H + H_2_O → OH + H_2_. J. Chem. Phys. 2011, 135, 084112 10.1063/1.3626525. 21895164

[ref46] Song H.; Guo H. Vibrational and Rotational Mode Specificity in the Cl + H_2_O → HCl + OH Reaction: A Quantum Dynamical Study. J. Phys. Chem. A 2015, 119, 6188–6194. 10.1021/acs.jpca.5b03740. 25988486

[ref47] Cheng Y.; Pan H.; Wang F.; Liu K. On the Signal Depletion Induced by Stretching Excitation of Methane in the Reaction with the F Atom. Phys. Chem. Chem. Phys. 2014, 16, 444–452. 10.1039/c3cp53036a. 24048150

[ref48] Meng F.; Yan W.; Wang D. Quantum Dynamics Study of the Cl + CH_4_ → HCl + CH_3_ Reaction: Reactive Resonance, Vibrational Excitation Reactivity, and Rate Constants. Phys. Chem. Chem. Phys. 2012, 14, 13656–13662. 10.1039/c2cp41917c. 22964797

[ref49] Pan H.; Cheng Y.; Liu K. Rotational Mode Specificity in Cl + CH_4_(*v*_3_=1,|*jNl*⟩): Role of Reactant’s Vibrational Angular Momentum. J. Phys. Chem. A 2016, 120, 4799–4804. 10.1021/acs.jpca.5b12156. 26761425

[ref50] Song H.; Li J.; Jiang B.; Yang M.; Lu Y.; Guo H. Effects of Reactant Rotation on the Dynamics of the OH + CH_4_ → H_2_O + CH_3_ Reaction: A Six-Dimensional Study. J. Chem. Phys. 2014, 140, 084307 10.1063/1.4866426. 24588169

[ref51] Zhang Z.; Zhang D. H. Effects of Reagent Rotational Excitation on the H + CHD_3_ → H_2_ + CD_3_ Reaction: A Seven Dimensional Time-Dependent Wave Packet Study. J. Chem. Phys. 2014, 141, 144309 10.1063/1.4897308. 25318724

[ref52] Liu R.; Wang F.; Jiang B.; Czakó G.; Yang M.; Liu K.; Guo H. Rotational Mode Specificity in the Cl + CHD_3_ → HCl + CD_3_ Reaction. J. Chem. Phys. 2014, 141, 074310 10.1063/1.4892598. 25149789

[ref53] Wang F.; Pan H.; Liu K. Imaging the Effects of Reactant Rotations on the Dynamics of the Cl + CHD_3_(*v*_1_ = 1, |*J,K*⟩) Reaction. J. Phys. Chem. A 2015, 119, 11983–11988. 10.1021/acs.jpca.5b03524. 26020295

[ref54] Czakó G. Quasiclassical Trajectory Study of the Rotational Mode Specificity in the O(^3^P) + CHD_3_(*v*_1_ = 0,1, *JK*) → OH + CD_3_ Reactions. J. Phys. Chem. A 2014, 118, 11683–11687. 10.1021/jp509891w. 25423322

[ref55] Szabó I.; Czakó G. Rotational Mode Specificity in the F^–^ + CH_3_Y [Y = F and Cl] S_N_2 reactions. J. Phys. Chem. A 2015, 119, 12231–12237. 10.1021/acs.jpca.5b06212. 26259068

[ref56] Papp P.; Czakó G. Rotational Mode Specificity in the F^–^ + CH_3_I(*v* = 0, *JK*) S_N_2 and Proton-Transfer Reactions. J. Phys. Chem. A 2020, 124, 8943–8948. 10.1021/acs.jpca.0c08043. 33054214 PMC7604870

[ref57] Yan P.; Meng F.; Wang Y.; Wang D. Energy Efficiency in Surmounting the Central Energy Barrier: a Quantum Dynamics Study of the OH + CH_3_ → O + CH_4_ Reaction. Phys. Chem. Chem. Phys. 2015, 17, 5187–5193. 10.1039/c4cp05488a. 25599813

[ref58] Yan P.; Wang Y.; Li Y.; Wang D. A Seven-Degree-of-Freedom, Time-Dependent Quantum Dynamics Study on the Energy Efficiency in Surmounting the Central Energy Barrier of the OH + CH_3_ → O + CH_4_ Reaction. J. Chem. Phys. 2015, 142, 164303 10.1063/1.4918981. 25933760

[ref59] Papp D.; Tajti V.; Győri T.; Czakó G. Theory Finally Agrees with Experiment for the Dynamics of the Cl + C_2_H_6_ Reaction. J. Phys. Chem. Lett. 2020, 11, 4762–4767. 10.1021/acs.jpclett.0c01263. 32441943 PMC7309313

[ref60] Hase W. L. Encyclopedia of Computational Chemistry; Wiley: New York, 1998; pp 399–407.

[ref61] Xie Z.; Bowman J. M. Permutationally Invariant Polynomial Basis for Molecular Energy Surface Fitting via Monomial Symmetrization. J. Chem. Theory Comput. 2010, 6, 26–34. 10.1021/ct9004917. 26614316

[ref62] Győri T.; Czakó G. Automating the Development of High-Dimensional Reactive Potential Energy Surfaces with the Robosurfer Program System. J. Chem. Theory Comput. 2020, 16, 51–66. 10.1021/acs.jctc.9b01006. 31851508

