# Supplementary material for: Rotational Mode-Specificity in the Cl + C2H6 → HCl + C2H5 Reaction
Source: J Phys Chem A. 2022 Apr 15;126(16):2551–60. doi: 10.1021/acs.jpca.2c01526 (PMC9059192; doi:10.1021/acs.jpca.2c01526)
Supplement: Supplementary file 1 — jp2c01526_si_001.pdf [file jp2c01526_si_001.pdf]

Supporting Information

Rotational Mode-Specificity in the Cl + C_2H_6 → HCl + C_2H_5 Reaction

Dóra Papp* and Gábor Czakó*

MTA-SZTE Lendület Computational Reaction Dynamics Research Group, Interdisciplinary Excellence Centre and Department of Physical Chemistry and Materials Science, Institute of Chemistry, University of Szeged, Rerrich Béla tér 1, Szeged H-6720, Hungary

*E-mail: dorapapp@chem.u-szeged.hu and gczako@chem.u-szeged.hu

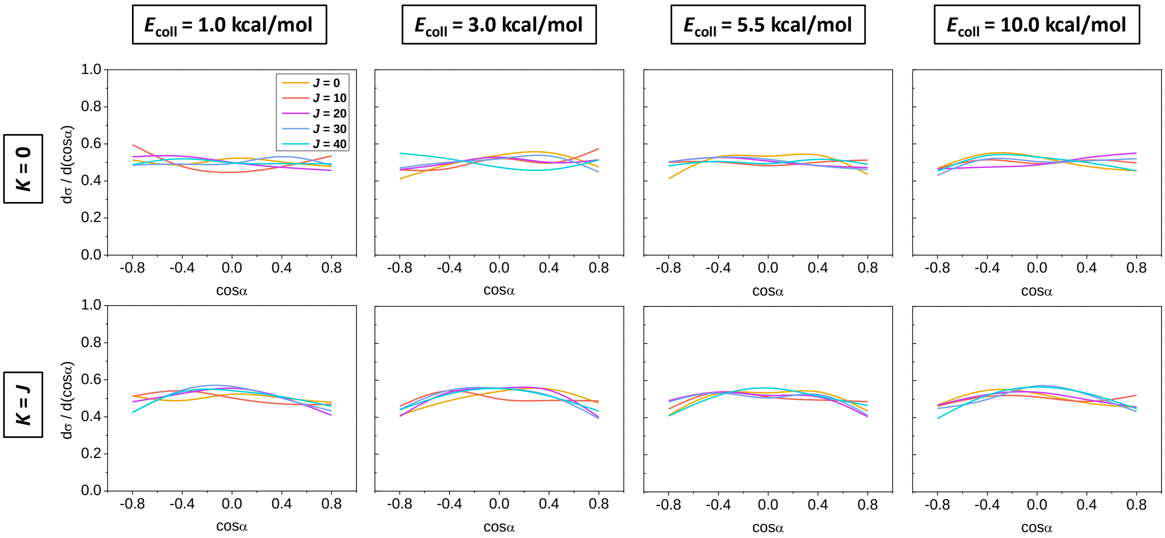

Figure S1 Normalized initial attack angle (the included angle of the initial C-C vector and the initial velocity vector of the ethane molecule) distributions of the reactants of the Cl + $C_2H_6(J, K)$ → HCl + C_2H_5 reaction in case of tumbling ($K = 0$) and spinning ($K = J$) rotational excitations for different J values at different collision energies.

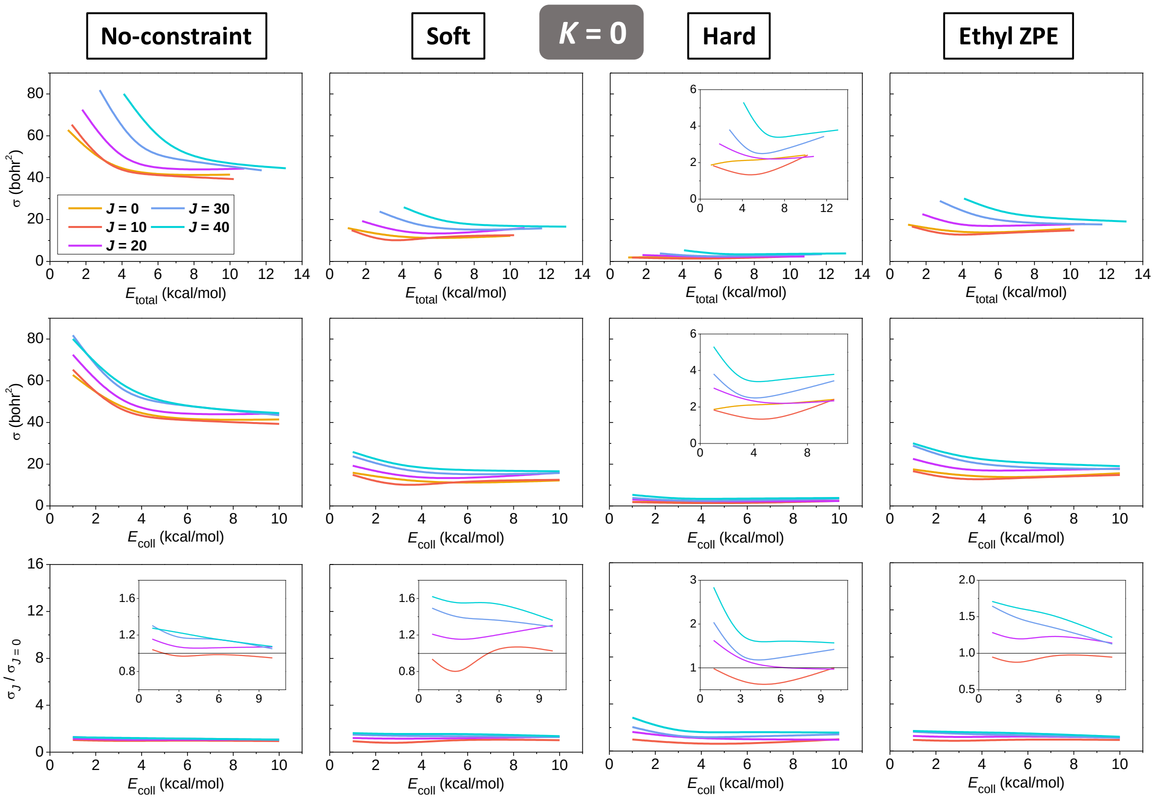

Figure S2 Integral cross sections (ICSs) of the Cl + C_2H_6(J, K = 0) → HCl + C_2H_5 reaction as a function of the total available initial energy (upper row), and the collision energy (middle row), and their enhancement factors relative to the ICSs of the rotationally unexcited reaction as a function of collision energy (lower row) applying different zero-point energy (ZPE) constraints for the products: (1) *soft*: the sum of the classical vibrational energy of the ethyl radical and the classical internal energy of the HCl product must be larger than ZPE(ethyl) + ZPE(HCl(J)), (2) *hard*: these constraints are set for each product separately, and (3) *ethyl ZPE*: the ZPE-constraint is only applied for C_2H_5.

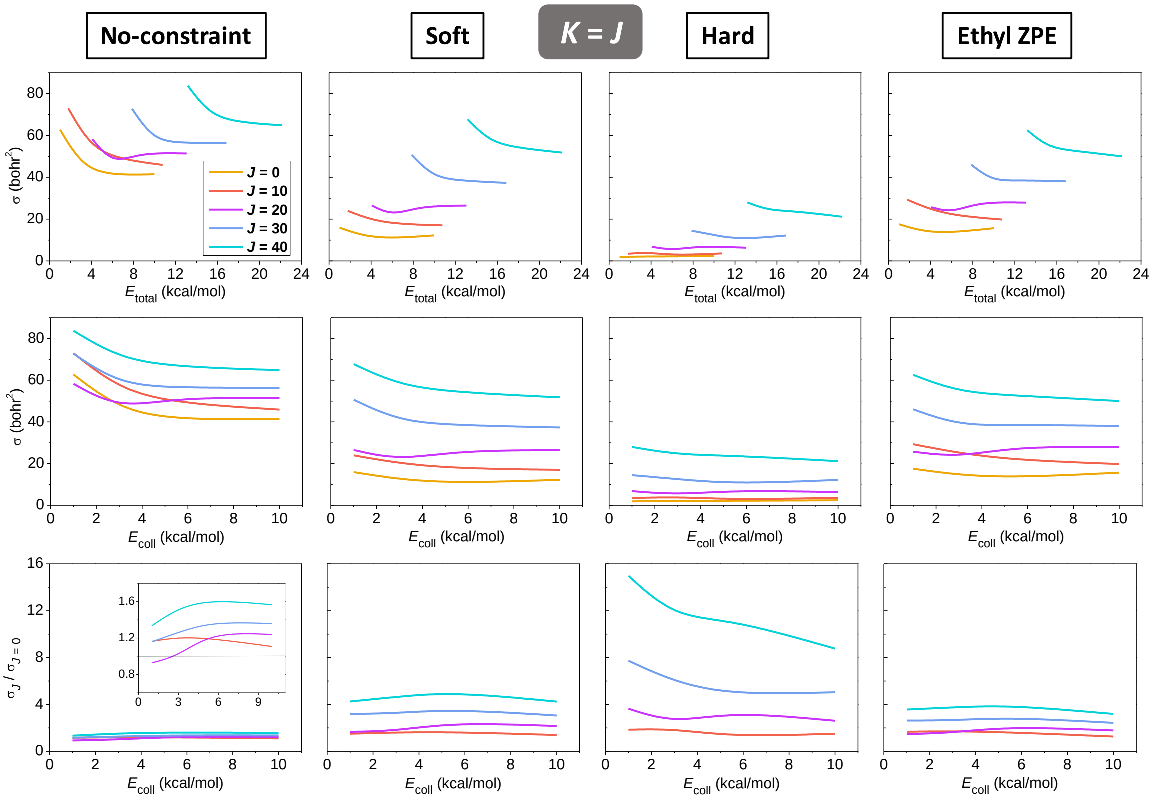

Figure S3 Integral cross sections (ICSs) of the Cl + C_2H_6(J, K = J) ⟶ HCl + C_2H_5 reaction as a function of the total available initial energy (upper row), and the collision energy (middle row), and their enhancement factors relative to the ICSs of the rotationally unexcited reaction as a function of collision energy (lower row) applying different zero-point energy (ZPE) constraints for the products: (1) *soft*: the sum of the classical vibrational energy of the ethyl radical and the classical internal energy of the HCl product must be larger than ZPE(ethyl) + ZPE(HCl(J)), (2) *hard*: these constraints are set for each product separately, and (3) *ethyl ZPE*: the ZPE-constraint is only applied for C_2H_5.
